# Dual-Task Training Interventions for Cerebral Palsy: A Systematic Review and Meta-Analysis of Effects on Postural Balance and Walking Speed

**DOI:** 10.3390/medicina61081415

**Published:** 2025-08-05

**Authors:** Irene Cortés-Pérez, María de los Ángeles Castillo-Pintor, Rocío Barrionuevo-Berzosa, Marina Piñar-Lara, Esteban Obrero-Gaitán, Héctor García-López

**Affiliations:** 1 Department of Health Sciences, University of Jaén, Campus Las Lagunillas s/n, 23071 Jaén, Spain; icortes@ujaen.es (I.C.-P.); rbb00009@red.ujaen.es (R.B.-B.); mpl00021@red.ujaen.es (M.P.-L.); 2 Faculty of Nursing, Physiotherapy and Medicine, University of Almería, Ctra. Sacramento s/n, 04180 La Cañada, Spain; maria.castiyo2002@gmail.com (M.d.l.Á.C.-P.); hector.garcia@ual.es (H.G.-L.); 3 CAIT APROMPSI Cazorla, C/Clara Campoamor 7, 23470 Cazorla, Spain

**Keywords:** cerebral palsy, dual-task training, motor and cognitive dual task, postural balance, gait, gross motor function

## Abstract

*Background and Objectives*: Dual-task training (DTT) is an innovative therapeutic approach that involves the simultaneous application of two tasks, which can be motor, cognitive, or a combination of both. Children with cerebral palsy (CP) often exhibit impairments in balance, motor skills, and gait, conditions that may be amenable to improvement through DTT. The aim of this study was to determine the effectiveness of DTT in enhancing balance, walking speed, and gross motor function-related balance in children with CP. *Materials and Methods*: In accordance with PRISMA guidelines, a comprehensive systematic review with meta-analysis (SRMA) was conducted. Electronic databases like PubMed Medline, Scopus, Web of Science, CINAHL, and PEDro were searched up to March 2025, with no language or publication date restrictions. Only randomized controlled trials (RCTs) examining the effectiveness of DTT on balance, gross motor function, and walking speed in children with CP were included. The methodological quality and risk of bias of the included RCTs were assessed using the PEDro scale. Pooled effects were calculated using Cohen’s standardized mean difference (SMD) and its 95% confidence interval (95% CI) within random-effects models. *Results*: Eight RCTs, providing data from 216 children, were included. Meta-analyses suggested that DTT was more effective than conventional therapies for increasing functional (SMD = 0.65; 95% CI 0.18 to 1.13), dynamic (SMD = 0.61; 95% CI 0.15 to 1.1), and static balance (SMD = 0.46; 95% CI 0.02 to 0.9), as well as standing (SMD = 0.75; 95% CI 0.31 to 1.18; *p* = 0.001) and locomotion dimensions (SMD = 0.65; 95% CI 0.22 to 1.08) of the Gross Motor Function Measure (GMFM) and walking speed (SMD = 0.46; 95% CI 0.06 to 0.87). Subgroup analyses revealed that a motor–cognitive dual task is better than a motor single task for functional, dynamic, and static balance and standing and locomotion dimensions for the GMFM. *Conclusions*: This SRMA, including the major number of RCTs to date, suggests that DTT is effective in increasing balance, walking and gross motor function-related balance in children with CP.

## 1. Introduction

Cerebral palsy (CP) comprises a group of neurodevelopmental and permanent disorders that primarily affect motor function and posture [1]. The multifactorial etiology of CP involves prenatal, perinatal, and postnatal factors, leading to non-progressive brain damage. This damage disrupts central nervous system development, particularly affecting the motor cortex and descending pathways, such as the corticospinal tract [2]. Recent data indicate that CP occurs in approximately 1.4 to 1.9 per 1000 live births [3,4]. The condition presents diverse clinical manifestations, including spasticity, muscle weakness, abnormal muscle tone, and difficulties in selective motor control [5]. These motor impairments profoundly affect the development of gross motor skills, including postural balance and gait, significantly limiting children’s activity and social participation [6,7].

Children with CP present with a complex interplay of sensory, motor, and cognitive deficits [8]. Specifically, they often exhibit alterations in the visual, somatosensory, and vestibular systems, which consequently diminish their balance skills and the generation of adaptive postural responses [9]. This leads to reduced anticipatory and reactive postural adjustments, thereby compromising the ability to proactively activate postural muscles and react effectively to perturbations [10]. Regarding gait skills, atypical patterns are commonly observed, characterized by reduced walking speed, shortened step length, increased base of support, and decreased cadence [11]. The combined limitations in balance and gait restrict the capacity of children with CP to explore their environment, interact with peers, and fully participate in daily activities [12]. Notably, walking speed is not only an indicator of functional capacity but has also been identified as a predictor of social participation in children with CP, with higher speeds correlating with greater engagement in recreational and community activities [13]. Therefore, it is crucial that interventions for children with CP are initiated as early as possible and are highly individualized. These interventions must consider each child’s unique motor and cognitive profile, as well as their family and social environment [14]. A comprehensive approach, integrating physiotherapy, occupational therapy, and psychological support, can significantly maximize the developmental potential and quality of life for children with CP [15].

Neuronal plasticity plays a critical role in the management of motor, sensory. and cognitive impairments in children with CP [16]. One example of rehabilitation programs based on neuroscientific principles is dual-task training interventions (DTT) [17]. Dual-task training (DTT) comprises the concurrent execution of two motor tasks with disparate objectives, or the integration of a motor and a cognitive task [18]. Rooted in the principle of training specificity, DTT simulates real-world demands, thereby facilitating simultaneous physical and cognitive rehabilitation for patients [19]. The effectiveness of DTT hinges on a patient’s capacity for motor task automaticity and their cognitive ability to concurrently manage diverse tasks [20]. The neurophysiological basis of DTT is rooted in “cognitive-motor interference” [21], where dividing attention and cognitive resources between simultaneous tasks increases demand on motor and cognitive control systems. This forces the brain to optimize its function and strengthen neural connections [22]. Neuronal plasticity can facilitate the integration of motor and sensory inputs, which are crucial to improving balance, coordination, and the execution of movements. DTT, by engaging the dorsolateral prefrontal cortex, anterior cingulate cortex, and cerebellum, can directly target brain areas crucial for attention, executive function, motor control, and balance [23,24]. In children with CP, where brain injury often disrupts these very networks, DTT’s ability to activate and potentially strengthen these connections offers a mechanism for functional improvement. To promote neuronal plasticity and improve the functional status of children with CP, repetitive tasks are essential for encouraging the brain to reinforce or create compensatory connections in dysfunctional areas [25]. Additionally, it is important to note that neuroplasticity is intimately related to age. Kachmar et al. (2019) identified a correlation between motor impairment and age, pointing to the potential for younger children with CP to achieve more substantial gains in motor skills [26]. This observation provides a strong rationale for prioritizing early interventions in this pediatric group.

The previous literature supports that DTT has shown promising results in improving balance, gait parameters, including walking speed, and gross motor function in patients with neurologic and neurocognitive disorders [27,28,29]. However, it is important to note the limited number of high-quality randomized controlled trials (RCTs) specifically in pediatric CP populations, suggesting a need for further rigorous research in this specific group to draw more definitive conclusions. Roostaei, M et al. (2021) conducted a systematic review with meta-analysis (SRMA) on children with CP [30]. This review, which included observational studies comparing children with CP to typically developing controls, suggested that DTT does not improve walking speed in children with CP. Despite the growing interest in DTT as a rehabilitation strategy for children with CP, to date, no SRMA, including only RCTs and children with CP, have assessed its effectiveness on balance, gait, and gross motor function. We hypothesize that DTT can improve balance, gross motor function, and gait skills of children with CP. To confirm this hypothesis and obtain robust, generalizable findings for this population, conducting a systematic review with meta-analysis (SRMA) that includes only randomized controlled trials (RCTs) with children with CP is the most appropriate approach. Therefore, the aim of this SRMA was to compile all available scientific evidence to date to assess the effectiveness of DTT in increasing functional, dynamic, and static balance, walking speed, and gross motor function-related balance and running and jumping skills in children with CP.

## 2. Materials and Methods

### 2.1. Study Design

This SRMA was conducted following the recommendations of the Preferred Reporting Items for Systematic Reviews and Meta-Analyses (PRISMA) 2020 statement [31] and the Cochrane Handbook for Systematic Reviews of Interventions [32]. The methodological quality of this SRMA was evaluated using the AMSTAR 2 checklist [33]. The protocol was previously registered in the PROSPERO database (CRD42025637875).

### 2.2. Literature Search and Bibliographical Sources

Two authors (I.C.-P. and M.A.C.-P.) independently conducted a comprehensive literature search from inception to March 2025, without publication date and language restrictions (to ensure including all potential studies), across electronic databases, including PubMed Medline, Scopus, Web of Science (WOS), CINAHL Complete, and PEDro (Physiotherapy Evidence Database). To minimize the risk of overlooking potentially eligible studies, the reference lists of retrieved references, conference proceedings, and abstracts were also manually screened. The search strategy was developed using the PICOS framework, focusing on the following elements [34]: Population (children with CP), Intervention (DTT), Comparison (conventional interventions or usual care), Outcomes (variables related to balance and gait skills) and Study Design (randomized controlled trials [RCTs] or pilot RCTs). To enhance search sensitivity and capture a wider range of relevant studies, the search strategy prioritized the “intervention” and “population” elements of the PICOS framework. Keywords employed in the search, informed by the MeSH thesaurus and other entry terms, included “cerebral palsy” and “dual-task training” as the most relevant (Supplementary Table S1). Boolean operators “AND” and “OR” were used to combine search terms effectively. A third author (E.O.-G.) with expertise in the field provided supervision throughout the literature search process.

### 2.3. Study Selection: Inclusion and Exclusion Criteria

Following the literature search, two authors (I.C.-P. and M.A.C.-P.) independently screened all retrieved studies by title and abstract. Cohen’s kappa coefficient (κ) was calculated to assess inter-rater agreement [35], with κ values interpreted as follows: poor (κ = 0), slight (0 < κ ≤ 0.20), fair (0.21 ≤ κ ≤ 0.40), moderate (0.41 ≤ κ ≤ 0.60), substantial (0.61 ≤ κ ≤ 0.80), and almost perfect (0.81 ≤ κ ≤ 1) [36]. A third author (EOG) resolved any discrepancies between reviewers. Studies were included in the meta-analysis if they met the following criteria that aligned with the PICOS: (1) Sample including children with CP; (2) DTT as experimental intervention; (3) conventional therapy or usual care as comparison intervention; (4) that variables providing statistical data related to balance and gait skills to include in the meta-analysis; and (5) RCTs or pilot RCTs. The exclusion criteria proposed were as follows: (1) studies including patients with CP and other neurological diseases in the same group; and (2) studies that did not report sufficient quantitative data for meta-analysis.

### 2.4. Data Extraction

Two authors (I.C.-P. and M.A.C.-P.) independently extracted data from the included studies using a standardized data extraction form. A third author (H.G.-L.) was consulted to resolve any disagreements. The following data were extracted: (1) study characteristics (authorship, year of publication, country, setting, funding source, and blinding status); (2) DDT characteristics (number of children per group, age, type of dual-task training, number of sessions, sessions per week, and duration of each session); (3) comparison intervention characteristics (type of intervention, number of sessions, sessions per week, and duration of each session); and (4) outcome measures (name of the variable, measurement tool used, mean and standard deviation of post-intervention scores, and time point of assessment).

### 2.5. Variables

In this SRMA the following outcomes were assessed: (1) Postural balance, differentiating on 3 specific dimensions: functional (ability to maintain postural balance during functional tasks), dynamic balance (ability to remain standing and stable during movement or displacement), and static (ability to maintain the body in position without movement or displacement). (2) Walking speed, defined as the rate at which steps are made while walking. (3) Gross motor function related to standing balance and walking, running, and jumping dimensions of the Gross Motor Function Measure (GMFM).

### 2.6. Assessment of the Methodological Quality, Risk of Bias, and Quality of Evidence

Two authors (R.B.-B. and M.P.-L.), independently, assessed the methodological quality of the included RCTs using the PEDro Scale, an 11-item scale designed to evaluate the methodological quality of clinical trials [37]. Total PEDro scores range from 0 to 10 (item 1 relates to external validity and is not included in the total score). Methodological quality was categorized as follows: excellent (9–10 points), good (6–8 points), moderate (4–5 points), and poor (≤3 points) [38]. PEDro scale items 2–3, 5–6, and 7 were used to assess the risk of selection, performance, and detection bias, respectively. Disagreements between the two reviewers were resolved through discussion with a third author (H.G.-L.).

The quality of evidence for each meta-analysis was assessed using the Grading of Recommendations Assessment, Development, and Evaluation (GRADE) assessment and the GRADE checklist by Meader et al. [39,40]. Five factors were considered: risk of bias, inconsistency, imprecision, indirectness of evidence, and publication bias. The quality of evidence was categorized as high, moderate, low, or very low, with downgrading by one level for each factor that was not adequately addressed.

### 2.7. Statistical Analysis

Two authors (E.O.-G. and I.C.-P.) conducted the meta-analysis using Comprehensive Meta-Analysis software (version 4, Biostat, Englewood, NJ, USA) [41]. Meta-analysis was only conducted if at least 2 RCTs provided data from an outcome. In all meta-analyses, Cohen’s standardized mean difference (SMD) with 95% confidence intervals (95% CI) was used as the effect size measure in a random-effects model [42,43]. The magnitude of the effect size was interpreted according to the guidelines proposed by Kinney et al. (2020) for rehabilitation studies: null (SMD = 0), small (SMD = 0.08–0.15), medium (SMD = 0.19–0.36), and large (SMD > 0.4) [44]. Forest plots were generated to visually represent the results of each meta-analysis [45]. In cases where studies reported the same outcome measured with the same instrument, the mean difference (MD) was additionally calculated to compare this finding with the minimal important difference (MID) for that measurement. Minimal important difference, previously known as minimally important clinical difference, is defined as “the smallest difference in score in the domain of interest which patients perceive as beneficial and which would mandate, in the absence of troublesome side effects and excessive cost, a change in patients’ management” [46]. This comparison allows us to determine the clinical relevance of the therapy assessed [47]. Publication bias was assessed using multiple methods, including funnel plot visualization [48], Egger’s test, and the trim-and-fill method [48,49,50]. The trim-and-fill method estimates the adjusted pooled effect size after accounting for potential publication bias [51]. If the difference between the original and adjusted pooled effect size exceeded 10%, the quality of evidence was downgraded by one level [52]. Heterogeneity across studies was assessed using the degree of inconsistency of Higgins (I^2^), the χ-square test, and its associated *p*-value [53,54]. Heterogeneity was categorized as follows: high (I^2^ > 50%), moderate (I^2^ 25–50%), low (I^2^ 5–25%), or trivial (I^2^ ≤ 5%) [55]. Additionally, a sensitivity analysis using the leave-one-out method was performed to evaluate the influence of individual studies on the overall pooled effect size. A subgroup analysis according to types of DTT intervention was conducted (motor–cognitive dual task vs. motor single task; motor–cognitive dual task vs. no task condition; and motor–motor dual task vs. motor single task).

## 3. Results

### 3.1. Study Selection

**Figure 1:**
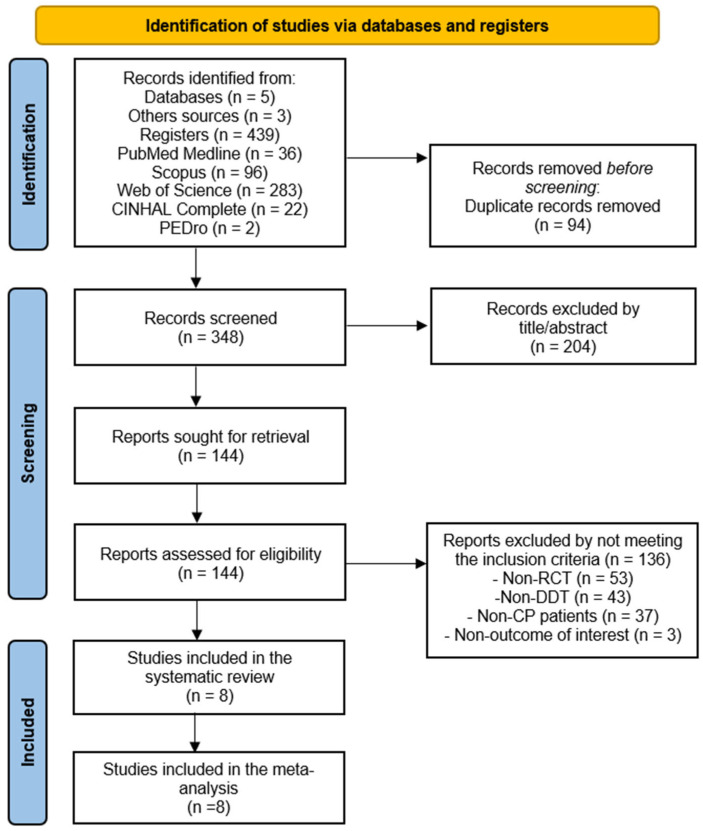
PRISMA flow diagram of the study selection process.

A total of 439 references were retrieved from the following databases: PubMed Medline (*n* = 36), Scopus (*n* = 96), Web of Science (*n* = 283), CINAHL Complete (*n* = 22), and PEDro (*n* = 2). An additional three references were identified through other sources, such as the reference lists. After removing 94 duplicates, 348 studies were screened by title and abstract. Firstly, 204 references were excluded for not being relevant by title/abstract and, later, 136 studies for not meeting the inclusion criteria (non-RCT *n* = 53, non-DTT *n* = 43, non-children with CP *n* = 37, and non-outcomes of interest *n* = 3). Finally, eight RCTs were included in the present SRMA [56,57,58,59,60,61,62,63]. The inter-rater agreement in the study selection process was excellent (κ = 0.91). The study selection process is summarized in the PRISMA flow diagram (Figure 1).

### 3.2. Characteristics of the Studies Included in the Review

The included RCTs, conducted between 2020 and 2024 in Egypt [56], China [63], Pakistan [57], Lithuania [58], South Korea [59], Saudi Arabia [60], Canada [61], and Turkey [62], enrolled 216 children with CP and a mean age of 8.2 ± 1.7 years old. The intervention group comprised 109 children, all of whom received DTT. Participants in this systematic review and meta-analysis (SRMA) exhibited a GMFCS level between I and III. Regarding the study comparisons, five studies contrasted motor–cognitive dual tasks with motor single tasks, two randomized controlled trials (RCTs) contrasted motor–motor dual tasks with motor single tasks, and a single RCT contrasted motor–cognitive dual tasks with a no-task condition. DTT interventions ranged from 3 to 12 weeks, including sessions ranging from 15 to 36 sessions, lasting between 15 and 50 min per session. The comparison group, consisting of 107 children, received conventional physiotherapy (treadmill training, vestibular training, or balance, gait, and lower limb exercises) [56,57,58,59,61,62,63], or children followed their daily living activities (they did not receive intervention) [60]. Control interventions were conducted between 3 and 12 weeks, 15–36 sessions, 2–5 sessions per week, and 30–50 min per session. The variables assessed in this meta-analysis were related to balance and gait skills. On the one hand, we assessed the following dimensions of the balance: functional, dynamic, and static balance. Related to gait skills, we assessed the walking speed. Additionally, in relation to these variables, the gross motor function related to balance and standing, running, and jumping abilities was evaluated. From all outcomes, the RCTs included provided data immediately post-intervention. Only one study [63] reported receiving external funding to conduct the research. Table 1 summarizes the characteristics of the included studies.

### 3.3. Methodological Quality and Risk of Bias of the Studies in the Review

The mean methodological quality of the RCTs included was good, showing a mean score in the PEDro Scale of 6.9 ± 0.6 points. The score of two RCTs [61,62] was confirmed on the PEDro website. The PEDro score of all RCTs ranged from six to eight points. The overall risk of bias among these studies was assessed as medium. Selection bias was present in three RCTs due to inadequate concealed allocation (item 3 was not met) [58,59,62]. Performance bias was also present in all RCTs, due to the nature of the intervention prevented blinding of participants and therapists (items 5 and 6 were not met). The score obtained from two studies was confirmed in the PEDro database. A detailed assessment of PEDro scores and reported biases for each individual study is presented in Table 2.

### 3.4. Meta-Analyses

The results of the meta-analyses, including the GRADE assessment, are summarized in Table 3.

#### 3.4.1. Functional Balance

**Figure 2:**
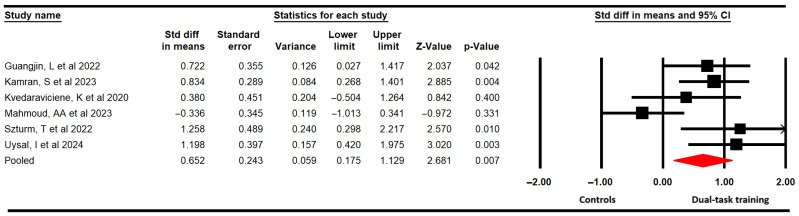
Forest plot of the effect of DTT on functional balance [56,57,58,61,62,63].

Six studies [56,57,58,61,62,63] with six independent comparisons provided data from 190 participants (31.7 per study) to assess the effectiveness of DTT on functional balance through the Pediatric Balance Scale (PBS). The meta-analysis showed that low-quality evidence of a large effect (SMD = 0.65; 95% CI 0.18 to 1.13; *p* = 0.007; I^2^ = 0%; χ = 4.92; df = 5; *p* = 0.43) favors DTT in increasing functional balance (Figure 2) without risk of publication bias (Egger *p* = 0.71). Furthermore, the meta-analysis revealed that DTT could increase 3.5 points (95% CI 1.47 to 5.41; *p* = 0.001; I^2^ = 16.8%; χ = 6.1; df = 5; *p* = 0.29) the score in the PBS (Supplementary Figure S1).

Subgroup analysis revealed that the motor–cognitive dual task is more effective than only the motor single task (SMD = 0.94; 95% CI 0.59 to 1.3; *p* < 0.001; I^2^ = 0%; χ = 1.35; df = 3; *p* = 0.71). However, non-statistically significant differences were found between the motor–motor dual task and motor single task (SMD = −0.04; 95% CI −0.72 to 0.66; *p* < 0.001; I^2^ = 40.5%; χ = 1; df = 1; *p* = 0.32).

#### 3.4.2. Dynamic Balance

**Figure 3:**
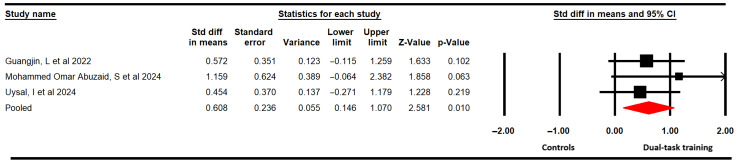
Forest plot of the effect of DTT on dynamic balance [60,62,63].

The effectiveness of DTT on dynamic balance was assessed, including three studies [60,62,63] with three independent comparisons that provided data from 76 participants (25.3 per study) using the Timed Up and Go Test (TUG). Our findings showed low-quality evidence that DTT is largely effective (SMD = 0.61; 95% CI 0.15 to 1.1; *p* = 0.01; I^2^ = 0%; χ = 0.96; df = 2; *p* = 0.62) in improving dynamic balance (Figure 3) without risk of publication bias (Egger *p* = 0.31). Additionally, the meta-analysis revealed that DTT can reduce 1.45 points (95% CI 0.15 to 2.76; *p* = 0.029; I^2^ = 10.7%; χ = 2.24; df = 2; *p* = 0.32) in the score in the TUG (Supplementary Figure S2).

Subgroup analysis revealed that the motor–cognitive dual-task is more effective than only the motor single task (SMD = 0.52; 95% CI 0.02 to 1.1; *p* = 0.042; I^2^ = 0%; χ = 0.05; df = 1; *p* = 0.82).

#### 3.4.3. Static Balance

**Figure 4:**
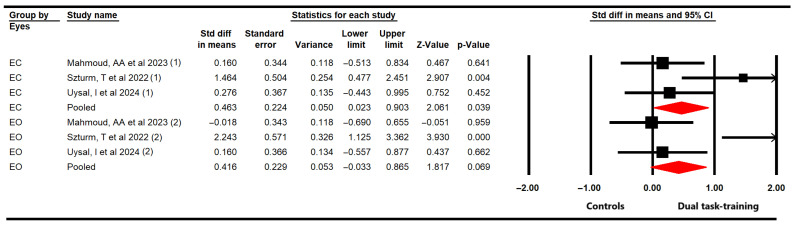
Forest plot of the effect of DTT on static balance while in eyes open and eyes closed conditions [56,61,62].

The effectiveness of DTT on static balance was assessed in eyes closed (EC) and eyes open (EO) conditions including three studies [56,61,62] with three independent comparisons in the meta-analysis of each condition. These studies provided data from 84 participants (28 per study) using posturographical tests. The meta-analysis revealed, with very low-quality evidence, only statistically significant differences in improving static balance in EC condition (SMD = 0.46; 95% CI 0.02 to 0.9; *p* = 0.039; I^2^ = 49,7%; χ = 5; df = 2; *p* = 0.08), and not in EO condition (SMD = 0.42; 95% CI −0.03 to 0.87; *p* = 0.069; I^2^ = 73.8%; χ = 12.4; df = 2; *p* < 0.001) (Figure 4). No risk of publication was found in any condition.

Subgroup analysis revealed that the motor–cognitive dual task is more effective than only the motor single task during EC (SMD = 0.64; 95% CI 0.11 to 1.27; *p* = 0.02; I^2^ = 55.5%; χ = 3.53; df = 1; *p* = 0.06).

#### 3.4.4. Gross Motor Function Related to Standing Balance and Walking, Running, and Jumping Abilities

**Figure 5:**
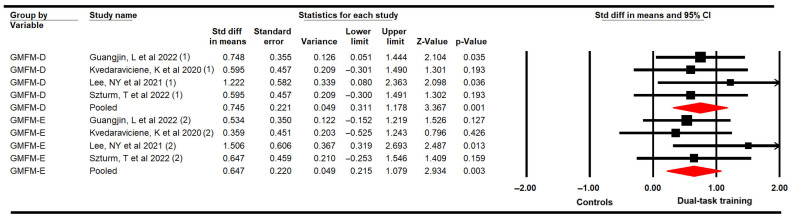
Forest plot of the effect of DTT on Gross Motor Function Measure D and E dimensions [58,59,61,63].

The effectiveness of DTT in improving standing (D) and walking, running, and jumping (e) dimensions of the Gross Motor Function Measure (GMFM) was assessed, including four studies [58,59,61,63] with four independent comparisons that provided data from 88 participants (22 per study) for each GMFM dimension. Our meta-analysis showed that DTT is largely effective in increasing standing (GMFM-D) (SMD = 0.75; 95% CI 0.31 to 1.18; *p* = 0.001; I^2^ = 0%; χ = 0.89; df = 3; *p* = 0.83) with low-quality evidence and walking, running, and jumping (GMFM-E) dimensions (SMD = 0.65; 95% CI 0.22 to 1.08; *p* = 0.003; I^2^ = 0%; χ = 2.53; df = 3; *p* = 0.47) (Figure 5) with very low-quality evidence. Additionally, the meta-analysis revealed that DTT can improve 1.9 points (95% CI 0.77 to 2.98; *p* = 0.001; I^2^ = 0%; χ = 2.49; df = 3; *p* = 0.48) and 3.8 points (95% CI 1.7 to 5.9; *p* < 0.001; I^2^ = 0%; χ = 0.56; df = 3; *p* = 0.91) the standing and walking, running and jumping dimensions, respectively, in the GMFM (Supplementary Figure S3). Risk of publication was present in the meta-analysis from the GMFM-E dimension (Egger *p* = 0.11). Trim-and-fill estimation reported an adjusted effect size of 14% minor (adjusted SMD = 0.52; 95% CI 0.35 to 0.12) than the original, revealing that publication bias was overestimating the true effect of DTT for this dimension (Supplementary Figure S4).

Subgroup analysis revealed that the motor–cognitive dual task is more effective than only the motor single task for GMFM-D (SMD = 0.79; 95% CI 0.28 to 1.29; *p* = 0.002; I^2^ = 0%; χ = 0.74; df = 2; *p* = 0.69) and for GMFM-E dimensions (SMD = 0.74; 95% CI 0.24 to 1.23; *p* = 0.004; I^2^ = 0%; χ = 2; df = 2; *p* = 0.37).

#### 3.4.5. Walking Speed

**Figure 6:**
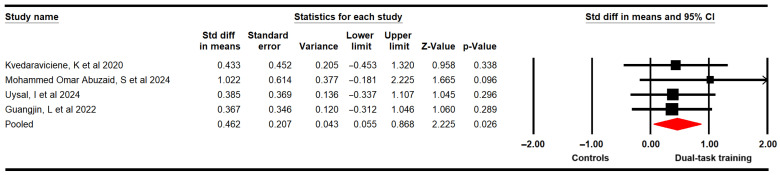
Forest plot of the effect of DTT on walking speed [58,60,62,63].

Four studies [58,60,62,63] with four independent comparisons provided data from 96 participants (24 per study) using the following tests: the 1 Minute Walk Test [58], the 10 Meters Walking Test [60], the 3 Meters Backward Walk Test [62], and the Maximum Speed Walking Test [63]. Our findings reveal very low-quality evidence of a large effect (SMD = 0.46; 95% CI 0.06 to 0.87; *p* = 0.026; I^2^ = 0%; χ = 0.96; df = 3; *p* = 0.81) of DTT in increasing waking speed (Figure 6). These findings can be influenced by the risk of publication bias (Egger *p* = 0.03). Trim-and-fill estimation reported an adjusted effect size of 13% major (adjusted SMD = 0.52; 95% CI 0.17 to 0.86) than the original, revealing that publication bias was underestimating the true effect of DTT in increasing walking speed in these children (Supplementary Figure S5).

Subgroup analysis revealed non-statistically significant differences between the motor–cognitive dual task and motor single task (SMD = 0.38; 95% CI −0.12 to 0.87; *p* = 0.137; I^2^ = 0%; χ = 0.001; df = 1; *p* = 0.98).

## 4. Discussion

While DTT is a promising approach that has demonstrated effectiveness in improving balance and gait skills in various neurological and neurocognitive disorders, no SRMA has been conducted specifically in children with CP. Although the SRMA by Roostaei et al. (2021) presents promising and encouraging results regarding the use of DTT in children with CP for improving balance and gait compared to healthy controls, it is crucial to acknowledge its limitations [30]. By exclusively including observational studies, the quality of evidence is significantly reduced. This is due to an inherently higher risk of bias (selection, confounding, or classification biases) in such designs, which precludes establishing the effectiveness of DTT. To assess the effectiveness of an intervention, an SRMA that includes RCTs would be necessary. Furthermore, Roostaei et al. applied English language filters in their search strategy. This restriction reduces the likelihood of identifying and including all relevant published studies on the topic, thereby limiting the comprehensiveness of this review. To address these limitations and the imperative need to consolidate scientific evidence to recommend DTT as an effective therapeutic tool for improving balance and gait in this population, an SRMA including only RCTs must be conducted. Therefore, our SRMA aimed to analyze the effect of DTT on improving balance dimensions and walking speed in children with CP. We employed a sensitive search strategy to screen the scientific literature, which yielded eight unique RCTs published to date (2020–2024) that met our inclusion criteria [56,57,58,59,60,61,62,63]. These RCTs provided data from 216 children with CP, enabling us to perform a meta-analysis of the following outcomes: functional, dynamic, and static balance; walking speed; and gross motor function related to balance and running/jumping skills. Our SRMA reports that DTT seems to be effective for functional, dynamic, and static balance and walking speed in children with CP. These findings are promising for making recommendations in clinical practice.

The postural balance is one of the main outcomes assessed in this SRMA. The RCTs included provided data allowing evaluation of the effectiveness of DTT in three balance dimensions, functional, dynamic, and static balance, while also evaluating EO and EC. Firstly, our findings demonstrate a large effect of DTT in improving functional balance in these children. Like all RCTs included that assessed functional balance using the PBS [56,57,58,61,62,63], our meta-analysis reported that DTT could increase the score by 3.5 points in the PBS. However, although the effect size was large, these results should be interpreted with caution. This is because the MD for PBS did not exceed the MID for PBS, which is 5.83 points [64]. The observed improvement in functional balance may be attributed to the ability of DTT to challenge postural and attentional control systems, thereby promoting greater adaptation and efficiency in the execution of daily activities [65]. On dynamic balance, assessed with the TUG test, our SRMA indicated that DTT is largely effective in increasing it. Additionally, we calculated that DTT can reduce the performance of the TUG test, which is clinically relevant. Our meta-analysis revealed that DTT can reduce by 1.45 s the performance of TUG in a group of children with CP and GMFCS I-II-III. Hassani, S et al. (2013) reported that MID for TUG test ranged from 1.1 to 1.7, 0.7 to 1.2 and 1.2 to 1.9 for children with CP and GMFCS levels I, II, and III [66], respectively. All children included in this meta-analysis reported GMFCS levels I to III. Our findings could be clinically relevant for improving dynamic balance, due to our findings exceeding the MID for each GMFCS level assessed. These findings encourage clinicians to integrate DTT in their conventional approaches to increase balance in children with CP. Finally, our meta-analysis also demonstrated that DTT effectively improves static postural balance, as measured by posturographic instruments during the EC condition. Regarding the EO condition, no statistically significant differences were observed in the improvement of static balance. It is important to note, however, that the scarcity of included RCTs and the presence of considerable heterogeneity (due to differences between the RCTs included and the posturographic tests used) may impact these findings, rendering them subject to modification upon the incorporation of future research. As no prior SRMA has examined these specific outcomes, our postural balance findings cannot be directly compared with those of other SRMA studies. However, our results are consistent with findings observed in other neurological disorders where DTT has been evaluated, such as stroke [28], Parkinson’s disease [29], and multiple sclerosis [67], although they cannot be directly compared due to differences between CP and these neurological disorders.

This SRMA contributes to the growing body of evidence supporting the effectiveness of DTT in gross motor function in children with CP. The observed improvements in the D (standing) and E (walking/running/jumping) domains of the GMFM in children with CP can be promising. Storm FA et al. 2020 reported that MID ranges for the GMFM-D and GMFM-E dimensions were 0.8–5.2% and 0.3–4.9%, respectively [68]. The values obtained in our meta-analysis fall within these ranges for each GMFM dimension analyzed, suggesting that these results could be clinically relevant. However, further studies are needed to confirm these findings in future meta-analyses. Specifically, DTT appears to positively influence walking speed and cadence, reduce gait variability, and enhance both static and dynamic balance, all of which are fundamental skills underlying the GMFM D and E domains [59]. However, these findings can be taken into account with caution, especially for the GMFM-E dimension, because the publication bias risk could be overestimating the true effect of DTT. It is plausible that these changes stem from enhanced sensorimotor integration, improved attention and executive function, and DTT-induced neuroplasticity [16]. By requiring children to coordinate multiple tasks simultaneously, DTT might contribute to the optimization of motor control and gait planning, which in turn could lead to notable improvements in standing, walking, running, and jumping abilities. Collectively, these findings underscore the potential of DTT as a valuable therapeutic intervention for ameliorating gross motor function and promoting functional independence in children with CP.

Finally, walking speed, recognized as a fundamental indicator of health and physical function, is crucial to improve in this population [69]. Our meta-analysis revealed that DTT is largely effective for increasing walking speed in children with CP. Nevertheless, we must declare the heterogeneity in the walking assessment instruments employed within this meta-analysis. While the random-effects model did not indicate statistical heterogeneity, this variation might impact the generalizability of the findings. Like the previous variables, the absence of prior SRMAs, including RCTs, makes it difficult to compare our results. Roostaei et al. (2021) reported that, in the group of children with CP and healthy controls, walking speed was reduced in DTT [30], suggesting that DTT cannot be beneficial to this goal. Opposite to this, our findings suggest that DTT is effective in increasing walking speed. This positions our findings as the most robust scientific evidence to date, considering their limitations. While these findings align with improvements in walking speed observed in Parkinson’s disease patients [29], despite the inherent differences between the two neurological conditions, this concordance provides important support for recommending DTT to improve walking speed. It is postulated that DTT improves walking speed in children with CP through various mechanisms, including neuroplasticity processes. We hypothesize that DTT stimulates neural plasticity, facilitating brain reorganization and the formation of new connections that contribute to improved motor function [70]. Furthermore, by challenging the child to coordinate multiple tasks simultaneously, DTT could promote the optimization of motor control and walking planning [71]. Finally, the execution of dual tasks demands greater attention to multiple aspects of the task, which can lead to an improvement in concentration capacity and, therefore, in gait efficiency [72].

Related to recommendations for clinical practice, we suggest that DTT may be beneficial for children with CP, especially classified as GMFCS levels I, II, and III. To simultaneously carry out motor and cognitive tasks is highly transferable to real daily living activities of each day. Additionally, DTT can be combined with other innovative and emerging therapies in CP such as virtual reality or robotic therapy [73]. Additionally, it is crucial to highlight that the individualization of DTT is fundamental to maximizing its benefits. The selection of tasks, their complexity, and the intensity of training should be adjusted to the capabilities, needs, and goals of each patient [74]. The promising results of DTT in children with CP, considering the limitations of this SRMA, could broaden its application to other neurological conditions and populations. It is recommended to explore its use in pediatric disorders such as autism and ataxias, aiming to improve coordination and executive functions. In adults, DTT benefits patients with Parkinson’s disease [29], multiple sclerosis [67], and early dementias [75], addressing both motor and cognitive deficits. Furthermore, its potential in geriatric rehabilitation and fall prevention is significant [76].

Although these findings can be clinically relevant in increasing balance, gait, and gross motor function skills in children with CP, some limitations must be considered. Firstly, some limitations related to methodology are that some meta-analyses were conducted, including a relatively small number of RCTs comprising a low sample size that can negatively affect the quality of evidence and generalizability of these novel findings. However, the search strategy applied was able to retrieve all RCTs published to date without applying language and publication date restrictions. A second methodological limitation stems from the medium methodological quality of the included RCTs and the presence of selection and performance biases within them. Selection bias, often resulting from inadequately concealed allocation, can lead to imprecise effect size estimates. This significantly reduces confidence in the conclusions drawn from any meta-analysis incorporating these RCTs. Performance bias, which typically arises when it is impossible to blind participants and therapists, can cause either an overestimation or underestimation of results. This is often due to the beliefs and expectations of participants or the unintended influence of the therapists themselves [77,78]. Third, publication bias was identified in two meta-analyses (GMFM-E and walking speed). Trim-and-fill analysis revealed that this bias led to an overestimation of DTT’s true effect on GMFM-E improvement and an underestimation of its effect on walking speed. Consequently, these findings should be considered with prudence, awaiting future meta-analyses that include additional RCTs to mitigate this bias. While it is generally recommended to include at least 10 studies to confidently assess publication bias, the low number of RCTs included in these assessments can reduce the precision of publication bias analyses. Another limitation stemmed from the heterogeneity observed in the interventions, the level of GMFCS of children included, and their respective measurement instruments (mainly in walking speed assessment), including RCTs that can make comparison between studies difficult, although this is a common limitation in physiotherapy RCTs. To address these limitations, future research should adopt more rigorous and standardized methodological designs. Increasing sample sizes and homogenizing intervention protocols and outcome measures would enhance the strength of the evidence and facilitate evidence-based clinical decision making.

## 5. Conclusions

This is the first SRMA to assess the effectiveness of DTT on balance, gait, and gross motor function in children with CP. Considering the study heterogeneity, the meta-analyses reveal early and promising evidence reporting that DTT seems to be more effective than conventional therapies for improving functional, dynamic, and static balance in these children. Subgroup analyses suggest that motor–cognitive dual-task conditions may be more effective than only motor single-task conditions. Furthermore, DTT has demonstrated clear effectiveness in increasing walking speed and gross motor function related to standing balance, as well as walking, running, and jumping abilities in this population. It seems that these findings hold clinical relevance for physiotherapists, suggesting their potential inclusion in rehabilitation protocols for children with CP. However, the limited number of studies included for some outcomes may restrict the generalizability of the results, and future studies are needed to strengthen these results.

## Figures and Tables

**Table 1:** Characteristics of the studies included in this review.

Study			DTT Group	Control Group		Outcome and Test	Qualitative Findings in Individual Studies	
	Sample Characteristics (n, Age, Sex)	Specific Task Comparisons	DTT Intervention Characteristics	Sample Characteristics (n, Age, Sex Ratio)	Control Intervention Characteristics		Intra-Group Differences	Inter-Group Differences
Guangjin, L et al. 2022 (China) Single-blinded RCT Setting: Quindao Women and Children’s Hospital Funding: Yes. Quindao Medical Science Guidance Program Project (2020-WJZD130)	18 children Mean age: 4.5 ± 0.4 Sex: 6G:12B GMFCS: I-II	Motor–cognitive dual task vs. motor single task	Walking on a treadmill while performing a series of five distinct motor and cognitive tasks DTT type: Motor and cognitive dual task Application: 20 sessions, for 4 weeks, 5 per week, and 50 min per session	16 children Mean age: 4.6 ± 0.5 Sex: 6G:10B GMFCS: I-II	Conventional therapy (treadmill training) Control type: motor single-task Application: 20 sessions, for 4 weeks, 5 per week, and 50 min per session	Functional balance (PBS)	Statistically significant improvement in both groups	Non-statistically significant differences between groups (*p* > 0.05)
						Dynamic balance (TUG)	Statistically significant improvement in both groups	Statistically significant differences favor DTT groups (*p* = 0.028)
						Walking speed (MSWT)	Statistically significant improvement in both groups	Non-statistically significant differences between groups (*p* > 0.05)
						Gross motor function (GMFM D-E)	Statistically significant improvement in both groups	Non-statistically significant differences between groups (*p* > 0.05)
Kamran, S et al. 2023 (Pakistan) Single-blinded RCT Setting: Physiotherapy Department of Allama Iqbal Memorial Hospital Funding: NR	26 children Mean age: 8.6 ± 1.9 Sex: 11B:15B GMFCS: II-III	Motor–cognitive dual-task vs. motor single task	Walking on a treadmill while performing a series of five distinct motor or cognitive tasks (lasting 3 min) DTT type: Motor and cognitive dual task Application: 8 weeks and 15 min per session. Session per week not reported	26 children Mean age: 8.5 ± 2 Sex: 14B:12B GMFCS: II-III	Conventional therapy (exercises for balance and gait improvement) Control type: Motor single task Application: 8 weeks and 15 min per session. Session per week and minutes NR	Functional balance (PBS)	Statistically significant improvement in both groups	Statistically significant differences favor DTT groups (*p* = 0.001)
Kvedaravičienė, K et al. 2020 (Lithuanian) Single-blinded RCT Setting: Lithuanian Sport University Funding: No	10 children Mean age: 10.4 ± 1.2 Sex: NR GMFCS: I-II	Motor–motor dual task vs. motor single task	Walking or standing on an unstable surfacer while tasking other motor task DTT type: Motor and motor dual-task Application: 15 sessions, for 3 weeks, 5 per week and 40 min per session	10 children Mean age: 10.4 ± 1.2 Sex: NR GMFCS: I-II	Conventional therapy (basic physiotherapy approach) Control type: motor single-task Application: 15 sessions, for 3 weeks, 5 per week and 40 min per session	Functional balance (PBS)	Statistically significant improvement in both groups	Statistically significant differences favor DTT groups (*p* < 0.05)
						Walking speed (1MWT)	Statistically significant improvement in both groups	Statistically significant differences favor DTT groups (*p* < 0.05)
						Gross motor function (GMFM D-E)	Statistically significant improvement in both groups	NR
Lee, NY et al. 2021 (South Korea) Single-blinded RCT Setting: Pediatric physical therapy center Funding: No	7 children Mean age: 9.4 ± 2.1 Sex: 3G:4B GMFCS: I-III	Motor–cognitive dual task vs. motor single task	Task performance of controlling balance on an unstable support surface accompanied with other motor task DTT type: Motor and cognitive dual-task Application: 16 sessions, for 8 weeks, 2 per week and 30 min per session	7 children; Mean age: 9.4 ± 2.3 9.42 ± 2.29 years; Sex: 4G:3B GMFCS: I-III	Neurodevelopmental treatment Control type: motor single-task Application: 16 sessions, for 8 weeks, 2 per week and 30 min per session	Gross motor function (GMFM D-E)	Statistically significant differences favor DTT groups (*p* < 0.05)	Statistically significant difference favor DTT groups (*p* < 0.05)
Mahmoud, A et al. 2023 (Egypt) Single-blinded RCT Setting: Clinic of faculty of physical therapy Funding: NR	17 children Mean age: 7.7 ± 2.2 Sex: 7G:10B GMFCS: I-II	Motor–motor dual task vs. motor single task	Walking on balance board while performing a motor task. DTT type: Motor and motor dual task Application: 24 sessions, for 8 weeks, 3 per week and 30 min per session	17 children Mean age: 7.6 ± 1.7 Sex: 10G:7B GMFCS: I-II	Vestibular training (balance and walking traditional exercises) Control type: Motor single-task Application: 24 sessions, for 8 weeks, 3 per week, and 30 min per session	Functional balance (PBS)	Statistically significant improvement in both groups	Non-statistically significant differences between groups (*p* = 0.33)
						Static balance (EO and EC)	Statistically significant improvement in both groups	Non-statistically significant differences between groups (*p* > 0.05)
Mohammed Omar Abuzaid, S et al. 2024 (Saudi Arabia) Single-blinded RCT Setting: Taiba Educational City Funding: NR	6 children Mean age: 9.3 ± 1.4 Sex: NR GMFCS: I-II	Motor–cognitive dual task vs. no task	A dual-task paradigm involving a motor task and a cognitive task requiring participants to name animals. DTT type: Motor and cognitive dual task Application: 16 sessions, for 8 weeks, 2 per week, and 30 min per session	6 children Mean age: 9.3 ± 1.4 Sex: NR GMFCS: I-II	Usual care (did not receive intervention) Control type: No task	Dynamic balance (TUG)	Statistically significant improvement in both groups	Statistically significant differences favor DTT groups (*p* = 0.001)
						Walking speed (10MWT)	Statistically significant improvement in both groups	Statistically significant differences favor DTT groups (*p* = 0.001)
Szturm, T et al. 2022 (Canada) Single-blinded RCT Setting: Physiotherapy Outpatient Department of SMD College of Medical Sciences Funding: No	10 children Mean age: 6.3 ± 2.3 Sex: 3G:7M GMFCS: I-III	Motor–cognitive dual task vs. motor single task	A dual-task paradigm involving a motor task requiring balance exercises and a cognitive task utilizing interactive videogames DTT type: Motor and cognitive dual task Application: 36 sessions, for 12 weeks, 3 per week, and 45 min per session	10 children Mean age: 6.3 ± 2.3 Sex: 3G:7M GMFCS: I-III	Conventional therapy (balance exercise program) Control type: Motor single task Application: 16 sessions, for 12 weeks, 3 per week, and 45 min per session	Functional balance (PBS)	Statistically significant improvement in both groups	Statistically significant differences favor DTT groups (*p* < 0.05)
						Static balance (EO and EC)	Statistically significant improvement in both groups	Statistically significant differences favor DTT groups (*p* = 0.03)
						Gross motor function (GMFM D-E)	NR	Non-statistically significant differences between groups (*p* > 0.05)
Uysal, I et al. 2024 (Turkey) Single-blinded RCT Setting: Private Son Atilim Special Education and Rehabilitation Center. Funding: NR	15 children Mean age: 9.8 ± 2.6 Sex: 5G:10B GMFCS: I-II	Motor–cognitive dual task vs. motor single task	A dual-task paradigm involving a motor task requiring balance, walking, and training exercises added to a cognitive task DTT type: Motor and cognitive dual task Application: 36 sessions, for 12 weeks, 3 per week, and 30 min per session	15 children; Mean age: 9.7 ± 2.8 Sex: 5G:10B GMFCS: I-II	Conventional therapy (lower limb physical exercise) Control type: Motor single task Application: 36 sessions, for 12 weeks, 3 per week, and 30 min per session	Functional balance (PBS)	Statistically significant improvement in both groups	Statistically significant differences favor DTT groups (*p* < 0.001)
						Dynamic balance (TUG)	Statistically significant differences in DTT group	Statistically significant differences favor DTT groups (*p* < 0.001)
						Walking speed (3-MBWT)	Statistically significant improvement in both groups	Statistically significant differences favor DTT groups (*p* < 0.001)
						Static balance (EO and EC)	Statistically significant differences in DTT group	Statistically significant differences favor DTT groups (*p* < 0.001)

Abbreviations: DTT, dual-task training; N, sample size; RCT, randomized controlled trial; G, girls; B, boys; Y, years; M, months; NR, non-reported; *p*, *p*-value; PBS, Pediatric Balance Scale; TUG, Timed Up and Go Test; MSWT, Maximum Speed Walking Test; GMFM, Gross Motor Function Measure; 1MWT, 1 Minute Walk Test; 10MWT, 10 Meters Walking Test; EO, eyes open; EC, eyes closed; and 3MWT, 3 Meters Backward Walk Test.

**Table 2:** PEDro score of the studies included in this review.

Study	PEDro Items											Total	Quality	Biases
	i1	i2	i3	i4	i5	i6	i7	i8	i9	i10	i11			
Guangjin, L et al. 2022	Y	Y	Y	Y	N	N	Y	Y	N	Y	Y	7/10	Good	Performance
Kamran, S et al. 2023	Y	Y	Y	Y	N	N	Y	Y	N	Y	Y	7/10	Good	Performance
Kvedaravičienė, K et al. 2020	Y	Y	N	Y	N	N	Y	Y	N	Y	Y	6/10	Good	Selection and performance
Lee, NY et al. 2021	Y	Y	N	Y	N	N	Y	Y	N	Y	Y	6/10	Good	Selection and performance
Mohammed Omar Abuzaid, S et al. 2024	Y	Y	Y	Y	N	N	Y	Y	N	Y	Y	7/10	Good	Performance
Mahmoud, A et al. 2023	Y	Y	Y	Y	N	N	Y	Y	N	Y	Y	7/10	Good	Performance
Szturm, T et al. 2022 *	Y	Y	Y	Y	N	N	Y	Y	Y	Y	Y	8/10	Good	Performance
Uysal, I et al. 2024 *	N	Y	N	Y	N	N	Y	Y	Y	Y	Y	7/10	Good	Selection and performance

Abbreviations: i1: eligibility criteria; i2: random allocation; i3: concealed allocation; i4: baseline comparability; i5: blind subjects; i6: blind therapists; i7: blind assessors: i8. measures of at least one key outcome are obtained from more than 85% of the subjects initially allocated to groups; i9: intention-to-treat analysis; i10: between-group comparisons; i11: point estimates and variability; Y: yes; and N: no. Note: Eligibility criteria item does not contribute to total score. Note: Score of the studies marked with * is confirmed in PEDro database (www.pedro.org.au, accessed on 18 April 2025).

**Table 3:** Main findings from meta-analyses and GRADE assessment.

Variable	Findings Summary										Quality Evidence (GRADE)					
	Effect Size					Heterogeneity		Publication Bias								
	K	N	N_s_	SMD [95% CI]	*p*	*Q* (*df*)	I^2^ (*p*)	Egger *p*	Trim and Fill		Risk of Bias	Inc	Ind	Imp	Pub Bias	Evidence Strength
									Adj SMD	% var						
Functional balance	6	190	31.7	0.65 [0.18 to 0.13]	0.007	4.92 (5)	0% (0.43)	0.71	0.65	0%	Medium	No	No	Yes	No	Low
Dynamic balance	3	76	25.3	0.61 [0.15 to 1.1]	0.01	0.96 (2)	0% (0.62)	0.31	0.61	0%	Medium	No	No	Yes	No	Low
Static balance EC	3	84	28	0.46 [0.02 to 0.9]	0.039	5 (2)	50% (0.08)	0.43	0.46	0%	Medium	Yes	No	Yes	No	Very low
Static balance EO	3	84	28	0.42 [−0.03 to 0.87]	0.069	12.4 (2)	73.8% (<0.01)	0.32	0.42	0%	Medium	Yes	No	Yes	No	Very low
GMFM standing	4	88	22	0.75 [0.31 to 1.18]	0.001	0.89 (3)	0% (0.83)	0.23	0.75	0%	Medium	No	No	Yes	No	Low
GMFM walking, running, jumping	4	88	22	0.65 [0.22 to 1.08]	0.003	2.53 (3)	0% (0.47)	0.11	0.52	14%	Medium	No	No	Yes	Yes	Very low
Walking speed	4	96	24	0.46 [0.06 to 0.87]	0.026	0.96 (3)	0% (0.81)	0.03	0.52	13%	Medium	No	No	Yes	Yes	Very low

Abbreviations: K, number of studies (comparisons) per meta-analysis; N, number of participants included in each meta-analysis; N_s_, average number of participants per meta-analysis; SMD, Cohen’s standardized mean difference; 95% CI, 95% confidence interval; *p*, *p*-value; Q, Q-test; df, degree of freedom; I^2^, degree of inconsistency of Higgins; Adj, adjusted; % var; percentage of variation; Inc, inconsistency; Ind, indirectness; Imp, imprecision; Pub, publication; GRADE, Grading of Recommendations Assessment, Development, and Evaluation assessment; EC, eyes closed; EO, eyes open; and GMFM, Gross Motor Function Measure.

## Data Availability

Data will be made available upon reasonable request to the corresponding author.

## Supplementary Materials

The following supporting information can be downloaded at https://www.mdpi.com/article/10.3390/medicina61081415/s1, Table S1. Search strategies used in each database; Figure S1. Forest plot for functional balance (mean difference); Figure S2. Forest plot for dynamic balance (mean difference); Figure S3. Forest plot for GMFM-D and E dimensions (mean difference); Figure S4. Forest plot for GMFM-E dimension; and Figure S5. Forest plot for walking speed.

## Abbreviations

The following abbreviations are used in this manuscript:

CP	Cerebral palsy
DTT	Dual-task training
SRMA	Systematic review and meta-analysis
RCTs	Randomized controlled trials
SMD	Standardized mean difference
95% CI	95% confidence interval
PRISMA	Preferred Reporting Items for Systematic Reviews and Meta-analyses
PEDro	Physiotherapy Evidence Database
TUG	Timed Up and Go Test
PBS	Pediatric Balance Scale
GMFM	Gross Motor Function Measure
