# Supplementary material for: Dual-Task Training Interventions for Cerebral Palsy: A Systematic Review and Meta-Analysis of Effects on Postural Balance and Walking Speed
Source: Medicina (Kaunas). 2025 Aug 5;61(8):1415. doi: 10.3390/medicina61081415 (PMC12387913; doi:10.3390/medicina61081415)
Supplement: Supplementary file 1 [file medicina-61-01415-s001.zip › medicina-3749159-supplementary.pdf]

SUPPLEMENTARY FILES

Table S1. Search stratagies used in each database

DATABASE	SEARCH STRATEGY
**PubMed Medline**	(cerebral palsy[mh] or cerebral palsy[tiab] or spastic diplegia[tiab] or CP[tiab] or spastic cerebral palsy[tiab] or Cerebral palsy, spastic, diplegic[mh] or Cerebral palsy, spastic, diplegic[tiab] or Spastic diplegia cerebral palsy[tiab]) and (dual task[tiab] or dual-task training[tiab] or double task[tiab] or dual task gait training[tiab])
**SCOPUS**	TITLE-ABS-KEY(¨cerebral palsy¨ or ¨spastic diplegia¨ or ¨CP¨ or ¨spastic cerebral palsy¨ or ¨Cerebral palsy, spastic, diplegic¨ or ¨Spastic diplegia cerebral palsy¨) and TITLE-ABS-KEY(¨dual task¨ or ¨dual-task training¨ or ¨double task¨ or ¨dual task gait training¨)
**Web of Science**	TOPIC(*cerebral palsy* or *spastic diplegia* or *CP* or *spastic cerebral palsy* or *Cerebral palsy, spastic, diplegic* or *Spastic diplegia cerebral palsy*) and TOPIC(*dual task* or *dual-task training* or *double task* or *dual task gait training*)
**CINAHL Complete**	AB(cerebral palsy or spastic diplegia or CP or spastic cerebral palsy or Cerebral palsy, spastic, diplegic or Spastic diplegia cerebral palsy) and AB(dual task or dual-task training or double task or dual task gait training)
**PEDro**	Cerebral palsy and dual-task<br>Cerebral palsy and dual task

Study name	Difference in means	Standard error	Variance	Lower limit	Upper limit	Z-Value	p-Value
Guangjin, L et al 2022	3,310	1,574	2,479	0,224	6,396	2,102	0,036
Kamran, S et al 2023	4,000	1,330	1,768	1,394	6,606	3,008	0,003
Kvedaraviciene, K et al 2020	1,900	2,236	5,000	-2,483	6,283	0,850	0,395
Mahmoud, AA et al 2023	-2,580	2,637	6,951	-7,747	2,587	-0,979	0,328
Szturm, T et al 2022	5,000	1,778	3,161	1,515	8,485	2,812	0,005
Uysal, I et al 2024	6,460	1,970	3,880	2,600	10,320	3,280	0,001
Pooled	3,447	1,005	1,010	1,477	5,416	3,430	0,001

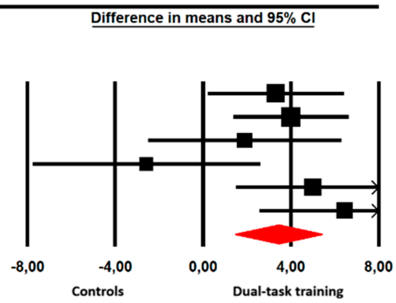

Figure S1. Forest plot for functional balance (mean difference)

Study name	Difference in means	Standard error	Variance	Lower limit	Upper limit	Z-Value	p-Value
Guangjin, L et al 2022	1,500	0,900	0,811	-0,265	3,265	1,666	0,096
Mohammed Omar Abuzaid, S et al 2024	4,990	2,486	6,178	0,118	9,862	2,008	0,045
Uysal, I et al 2024	0,950	0,764	0,583	-0,547	2,447	1,244	0,213
Pooled	1,453	0,665	0,442	0,150	2,757	2,185	0,029

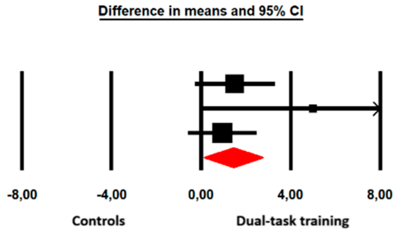

Figure S2. Forest plot for dynamic balance (mean difference)

SUPPLEMENTARY FILES

Group by Variable	Study name	Difference in means	Standard error	Variance	Lower limit	Upper limit	Z-Value	p-Value
GMFM-D	Guangjin, L et al 2022 (1)	1,430	0,657	0,432	0,142	2,718	2,177	0,030
GMFM-D	Kvedaraviciene, K et al 2020 (1)	2,500	1,880	3,536	-1,186	6,186	1,329	0,184
GMFM-D	Lee, NY et al 2021 (1)	4,570	2,000	3,998	0,651	8,489	2,286	0,022
GMFM-D	Szturm, T et al 2022 (1)	2,500	1,878	3,528	-1,181	6,181	1,331	0,183
GMFM-D	Pooled	1,874	0,565	0,319	0,767	2,981	3,317	0,001
GMFM-E	Guangjin, L et al 2022 (2)	3,640	2,344	5,495	-0,955	8,235	1,553	0,120
GMFM-E	Kvedaraviciene, K et al 2020 (2)	2,100	2,616	6,845	-3,028	7,228	0,803	0,422
GMFM-E	Lee, NY et al 2021 (2)	4,290	1,523	2,319	1,306	7,274	2,817	0,005
GMFM-E	Szturm, T et al 2022 (2)	4,300	2,974	8,845	-1,529	10,129	1,446	0,148
GMFM-E	Pooled	3,789	1,071	1,146	1,691	5,887	3,539	0,000

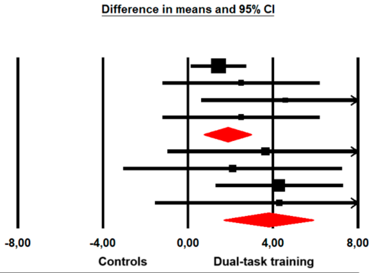

Figure S3. Forest plot for GMFM-D and E dimensions (mean difference)

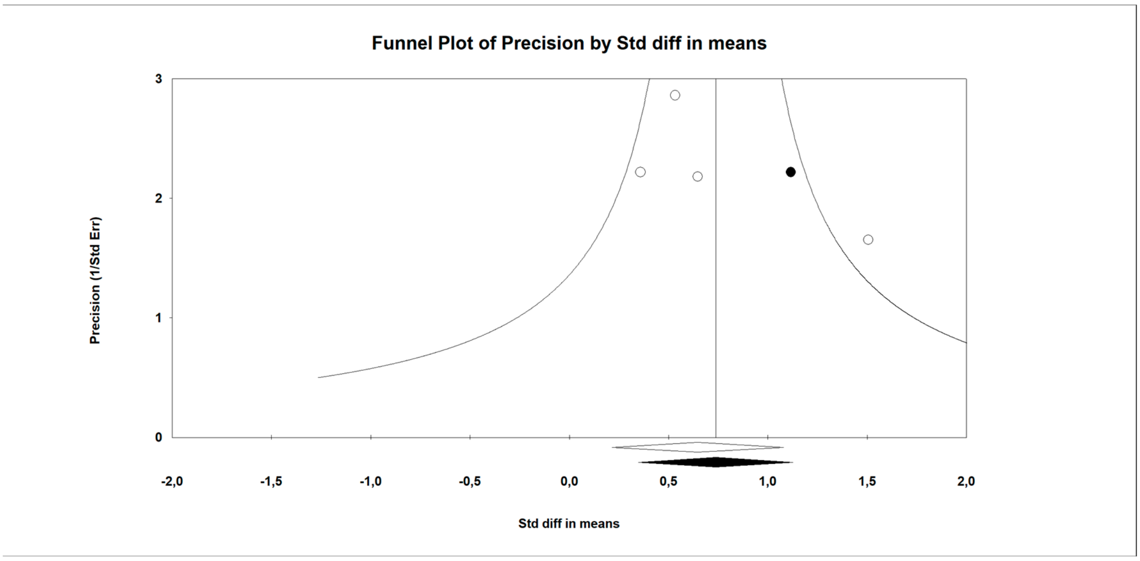

Figure S4. Forest plot for GMFM-E dimension

SUPPLEMENTARY FILES

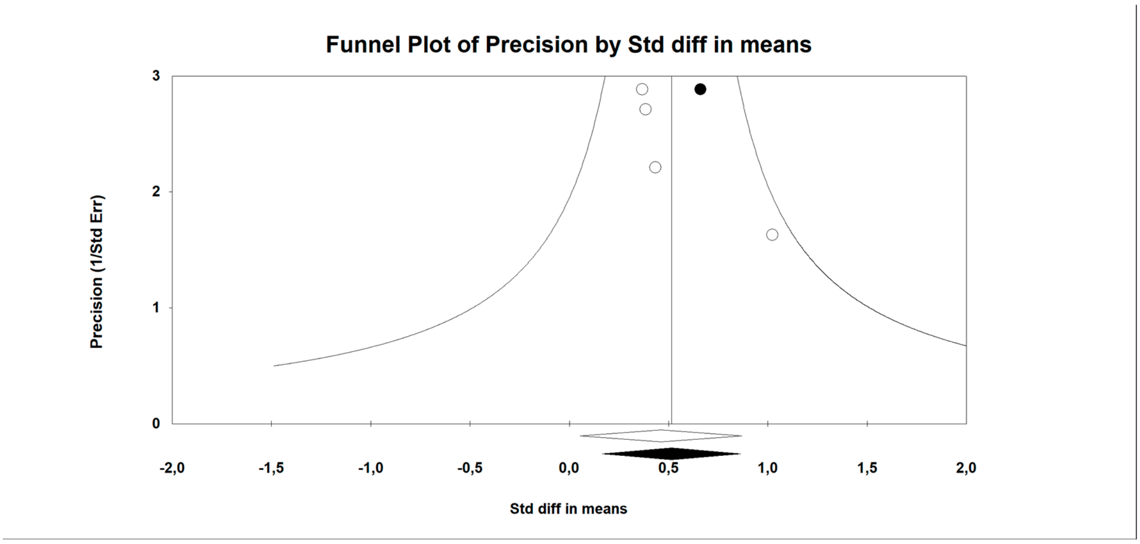

Figure S5. Forest plot for walking speed
